# Correction to “Exploring the Inner- and Outer-Sphere Mechanistic Pathways of ORR on M–N–Cs with Pyrrolic MN_4_ Motifs”

**DOI:** 10.1021/acs.jpcc.4c03957

**Published:** 2024-07-16

**Authors:** Jian Liang Low, Christina Roth, Beate Paulus

In the original publication of our article, there was a typographical error in eq 3. The power of −1 at the end of the equation was incorrect. The corrected equation is
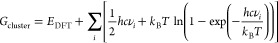
3We confirm that this typographical error does not affect the results or conclusions of the study. We apologize for any confusion this may have caused.

